# Monitoring of the Initial Stages of Diamond Growth on Aluminum Nitride Using In Situ Spectroscopic Ellipsometry

**DOI:** 10.1021/acsomega.3c03609

**Published:** 2023-08-11

**Authors:** William Leigh, Soumen Mandal, Jerome A. Cuenca, David Wallis, Alexander M. Hinz, Rachel A. Oliver, Evan L. H. Thomas, Oliver Williams

**Affiliations:** † School of Physics and Astronomy, Cardiff University, Cardiff CF24 3AA, U.K.; ‡ EPSRC Centre for Diamond Science and Technology, Coventry CV4 7AL, U.K.; § School of Engineering, Cardiff University, Cardiff CF10 3AT, U.K.; ∥ Department of Materials Science and Metallurgy, University of Cambridge, Cambridge CB3 0FS, U.K.

## Abstract

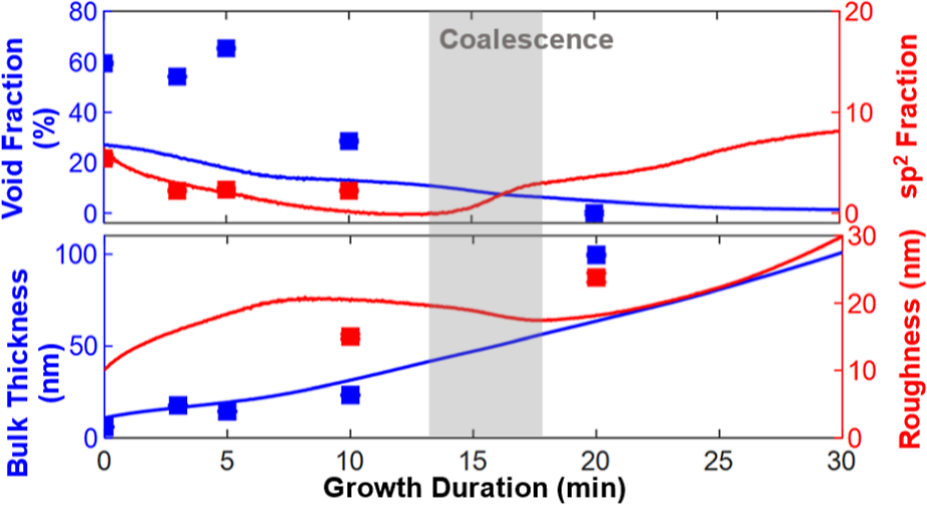

The high thermal conductivity of polycrystalline diamond makes it ideally suited for thermal management solutions for gallium nitride (GaN) devices, with a diamond layer grown on an aluminum nitride (AlN) interlayer atop the GaN stack. However, this application is limited by the thermal barrier at the interface between diamond and substrate, which has been associated with the transition region formed in the initial phases of growth. In this work, in situ spectroscopic ellipsometry (SE) is employed to monitor early-stage microwave plasma-enhanced chemical vapor deposition diamond growth on AlN. An optical model was developed from ex situ spectra and applied to spectra taken in situ during growth. Coalescence of separate islands into a single film was marked by a reduction in bulk void fraction prior to a spike in sp^2^ fraction due to grain boundary formation. Parameters determined by the SE model were corroborated using Raman spectroscopy and atomic force microscopy.

## 1 Introduction

Gallium nitride (GaN) is a promising material for high-electron-mobility transistors in high-frequency and high-power applications.^1−3^ However, an increase in the operating temperature of these devices results in a significant reduction in device lifetime.^4,5^ Presently, temperature management solutions involve the manufacture of devices from GaN grown atop silicon carbide (SiC),^6^ which has a thermal conductivity of between 360 and 490 W/m K.^7^ The thermal conductivity of polycrystalline diamond is significantly higher at approximately 1200 W/m K for a 100 μm thick layer.^8^ While the growth of thick diamond layers directly on GaN is very challenging,^9^ the successful growth of thick diamond layers on aluminum nitride (AlN) has been demonstrated,^10^ opening the possibility of diamond growth on an AlN layer atop the GaN stack.^11,12^ A significant limitation to such use of diamond in thermal management applications is the interfacial thermal barrier between diamond film and substrate. The nature of the interfacial region of the diamond film has a significant impact on the thermal properties of the interface. Polycrystalline diamond films with a thicker defective region at the interface have been shown to display increased thermal barrier resistance.^13^ The thermal conductivity of polycrystalline diamond films decreases with an increasing level of non-diamond sp^2^ content within the film.^14,15^ As the level of sp^2^ content incorporated in the film is heavily dependent on growth conditions,^16−18^ in situ monitoring is necessary to identify growth conditions that result in low levels of sp^2^ within the film while minimizing the thickness of the defective interfacial region.

Electron-based techniques are impractical for monitoring microwave plasma-enhanced chemical vapor deposition (MPECVD) diamond growth due to the pressures involved, and the plasma environment mostly limits monitoring to optical techniques such as pyrometric and laser interferometry.^19,20^ While these techniques are useful for monitoring the bulk film growth, they are limited in resolution and are unable to determine the structure and composition of films. In comparison, spectroscopic ellipsometry (SE) has the ability to identify the structure, thickness, and composition of diamond films as thin as 4 nm.^21^ The technique works by measuring the changes in polarization of light after reflection from a sample, with measured spectra compared to simulated spectra produced by an optical model. In the fitting process, model parameters are selected to be allowed to vary to reduce the mean square error (MSE) between measured and modeled spectra.^22^ As SE relies on the measurement of a change in polarization and not simply intensity, it can be used even when a bright plasma background is present.^23^ Previous SE studies of polycrystalline diamond films have utilized a Bruggeman effective medium approximation (EMA) to model the diamond layer, mixing known optical constants of void, diamond, and sp^2^ material.^16,17,21,24−26^

## 2 Materials and Methods

The AlN layer was grown on a 150 mm silicon substrate by metal organic vapor-phase epitaxy (MOVPE) using an Aixtron 1 × 6″ close-coupled shower head reactor. The Si substrate was first annealed at a high temperature (approximately 1070 °C) to remove the native oxide and then exposed to a brief NH_3_ flux to nitridate the Si surface. AlN growth was then initiated for 660 s at a temperature of 960 °C using trimethyl aluminum as a precursor in a H_2_ carrier gas before the temperature was increased to approximately 1100 °C for the remainder of the AlN growth. The reactor pressure was 50 mbar during the AlN growth.

The total thickness of the AlN layer was measured at approximately 180 nm using SE, with no significant variation seen between samples. The measured thickness is consistent with the expected thickness based on the design of the process used in MOVPE. Prior to diamond growth, samples were pre-treated for 10 min in a N_2_/H_2_ plasma as detailed in^10^ to improve the adherence of the diamond film to the AlN substrate. Substrates were seeded by immersing them in a nanodiamond/DI H_2_O colloid^27^ and placing them in an ultrasonic bath for 10 min, a technique previously shown to result in high seeding densities on aluminum nitride.^10^

**Figure 1:**
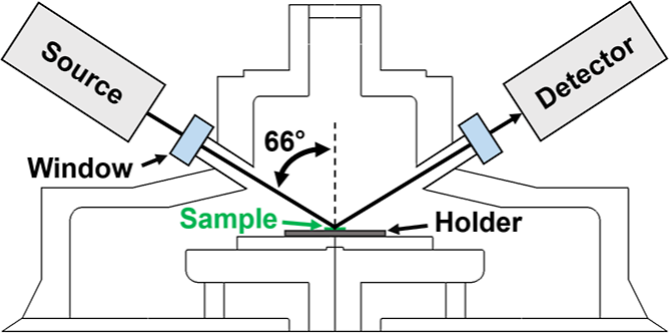
Setup used to measure in situ SE spectra. The sample thickness has been exaggerated for visibility.

Diamond films were grown in a Carat Systems CTS6U clamshell-type MPECVD reactor, with a chamber pressure of 50 Torr and microwave power of 3 kW, resulting in substrate temperatures of approximately 730 °C measured using a WilliamsonIR Pro92 dual-wavelength pyrometer. A gas flow of 3% methane in hydrogen was used for growth durations of 3–90 min. SE spectra were measured using a J. A. Woollam M-2000 rotating compensator ellipsometer over a wavelength range of 370–1000 nm. Ex situ spectra were measured at incidence angles of 75, 70, and 65°, with in situ spectra measured at an incidence angle of approximately 66° through fixed fused silica viewports. The in situ SE setup is shown in Figure 1. An iterative fitting process (described in Sections 1–3) in the CompleteEASE software was used to generate a SE model for sample characterization, with measured spectra compared with simulated spectra and potential sample structures and parameters varied to reduce the MSE between these. Atomic force microscopy (AFM) was performed using a Bruker Dimension Icon microscope equipped with a ScanAsyst tip operating in PeakForce Tapping mode. Raman spectra were measured with an excitation wavelength of 532 nm using a HORIBA LabRAM spectrometer.

## 3 Results and Discussion

### 3.1 Modeling AlN Layer

Given that the optical constants of AlN vary significantly with growth conditions,^28,29^ it is impractical to utilize reference optical constants to model this layer. Previous attempts at characterization of AlN films using spectroscopic ellipsometry have employed a Cauchy^30^ or Cauchy–Urbach^31^ dispersion to approximate the optical constants of the AlN layer. The Cauchy dispersion accounts for refractive index as a function of wavelength
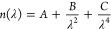
1where *n*(λ) is the refractive index at wavelength λ and *A*, *B*, and *C* are fitted model parameters. This model assumes that the modeled layer is transparent in the wavelength range used, with the extinction coefficient *k* assumed to be zero.

The Cauchy–Urbach model adds a second function to account for the absorption tail, with the extinction coefficient as a function of wavelength given by
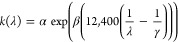
2where *k*(λ) is the extinction coefficient at wavelength λ, α and β are fit parameters, and γ is the band edge. These two models are only valid in the case of normal dispersion, where the refractive index increases with shorter wavelength.

**Figure 2:**
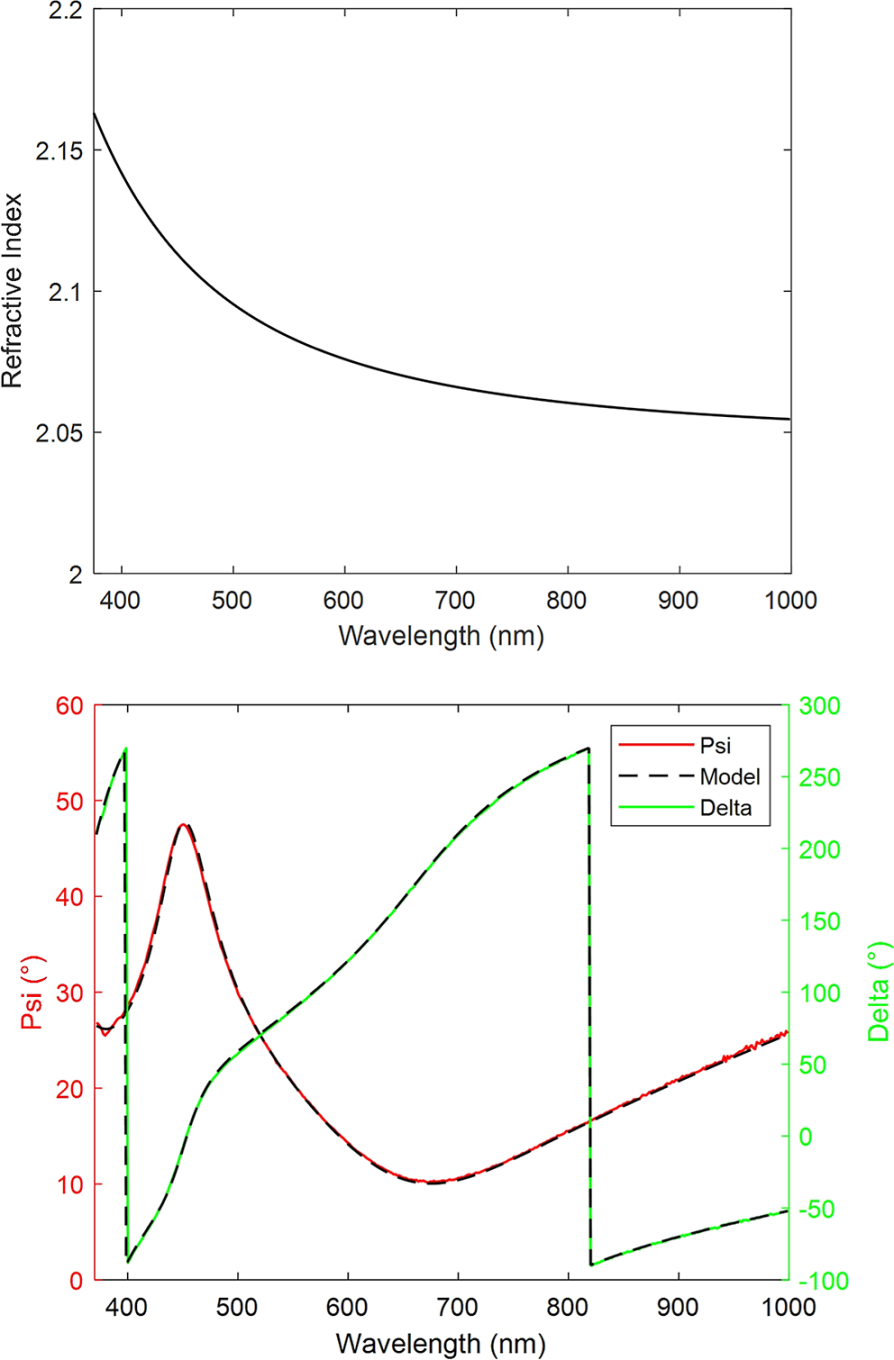
Above: Refractive index used to model the AlN layer. Below: Measured and modeled spectra of the post-treatment AlN sample with a 70° angle of incidence.

Ex situ spectra of an AlN sample after pre-treatment were used to produce an optical model of the AlN film. The first version of the model comprised a Cauchy layer on top of a silicon substrate,^32^ with the *A* and *B* parameters allowed to vary, resulting in a MSE of 14.804 between measured and modeled spectra. Also, allowing the *C* parameter to vary further reduced the MSE to 10.141. The addition of an Urbach term proved unnecessary in this case, as its inclusion did not change the MSE, with the magnitude of *k* suggested to be extremely low. Therefore, the Cauchy model alone was used to model the AlN film, with *k* assumed to be zero. Finally, MSE was reduced to 8.439, including a 1.82 nm thick layer consisting of 50% void and 50% bulk layer to approximate surface roughness. In the final version of the model, the AlN layer was 179.87 nm thick. Figure 2 shows the refractive index used to model the AlN layer, along with the measured and modeled SE spectra for this final version of the model, showing a good match between the measured and modeled spectra. The modeled trends in optical constants with wavelength are consistent with previously determined values of AlN samples.^33−35^

### 3.2 Ex Situ Model of the Diamond Film

**Figure 3:**
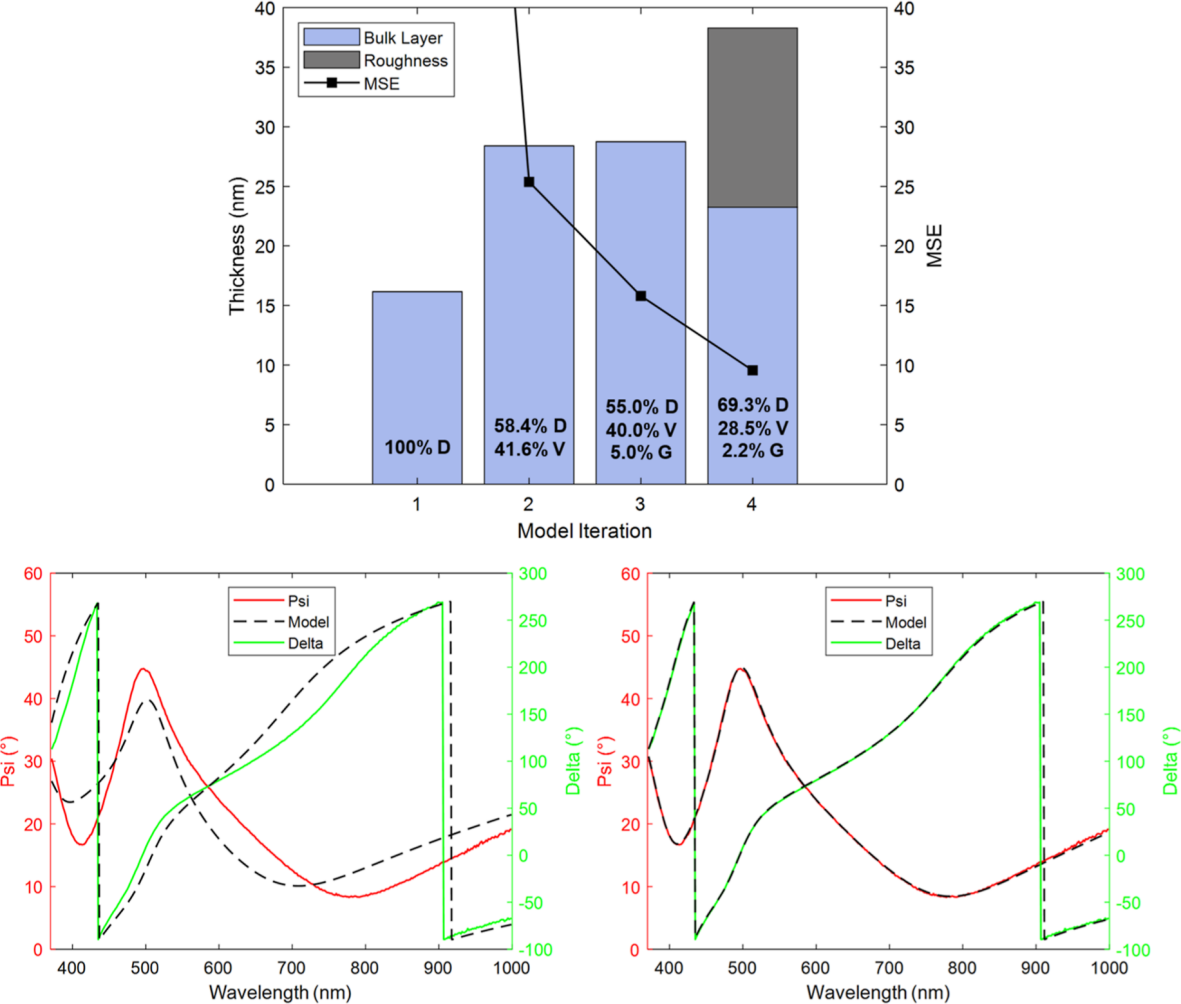
Above: Progression of the model over the fitting process, showing MSE, bulk layer thickness and void (V), diamond (D), and glassy carbon (G) content for the 10 min growth duration sample. Below: Comparison between measured and modeled spectra at an incidence angle of 70° for the initial (left) and final (right) models.

Ex situ spectra of a diamond sample grown for a duration of 10 min were used to produce an SE model. Figure 3 shows the variation of the model over the fitting process. The first version of this model comprised a silicon substrate^32^ with a 179.87 nm thick AlN layer modeled using the optical constants determined in Section 3.1. The diamond layer atop this was approximated using oscillators matched to the optical constants of type I and type II natural diamonds.^36^ The spectra simulated using this model significantly differed from the measured spectra, with MSE exceeding 140. A very large decrease in MSE to 25.380 was achieved by accounting for void content in the bulk layer. This was achieved by using an EMA containing both diamond and void content to approximate this layer, with the void content allowed to vary. MSE was further reduced to 15.796 by the addition of optical glassy carbon optical constants^37^ to account for sp^2^ content in the bulk layer. It has previously been shown that glassy carbon is an effective approximation to the model sp^2^ content in polycrystalline diamond films.^16,17,24,26^ Finally, surface roughness was accounted for with a second EMA atop the bulk layer consisting of 50% void and 50% bulk, resulting in a reduction of the MSE to 9.554. When applying the model to samples of growth duration between 3 and 20 min, as well as a seeded sample, it was found that the inclusion of the roughness layer was only necessary from the 10 min sample onward; its inclusion did not decrease the MSE of the 3 min sample and was rejected by the fitting process when fitting the 5 min and seeded samples. Applying the SE model to a sample grown for 20 min produced MSE in excess of 100 due to depolarization caused by the increased surface roughness resulting from crystallite overgrowth. The SE-derived bulk thicknesses of the ex situ samples are shown in Table 1.

**Table 1:** Diamond Film Thicknesses of the Ex Situ Samples

growth duration (min)	diamond film thickness (nm)
3	17.63
5	14.32
10	23.25
20	99.55

### 3.3 In Situ Fitting

The model was modified slightly to account for the changes introduced by the application to in situ data. In-plane window effects from the fused silica windows were accounted for by measuring the spectra of a reference sample as described in.^38^ A second consideration was the impact of the elevated temperature on the optical constants of the silicon substrate. These optical constants were replaced with those of silicon from a temperature-dependent library in the CompleteEASE software, with the temperature set to the substrate temperature of 730 °C measured using a dual-wavelength pyrometer. While the refractive index of the modeled AlN layer did vary slightly when the Cauchy parameters were allowed to vary when fitting the in situ data, this did not impact the trends seen in the modeled diamond layer thickness and composition. As a result, the Cauchy parameters were not allowed to vary in the in situ model to minimize the impact of any potential parameter correlation. Parameter correlation occurs during the data fitting process in cases where changes in multiple different parameters exhibit the same spectral signature, resulting in multiple combinations of parameter values producing an identical quality of fit, with no unique determination of optimal parameter values.^39^ Due to the fact that the refractive index of diamond films is not changed significantly by an increase in temperature,^40^ it was not necessary to modify the optical constants of the bulk layer components.

**Figure 4:**
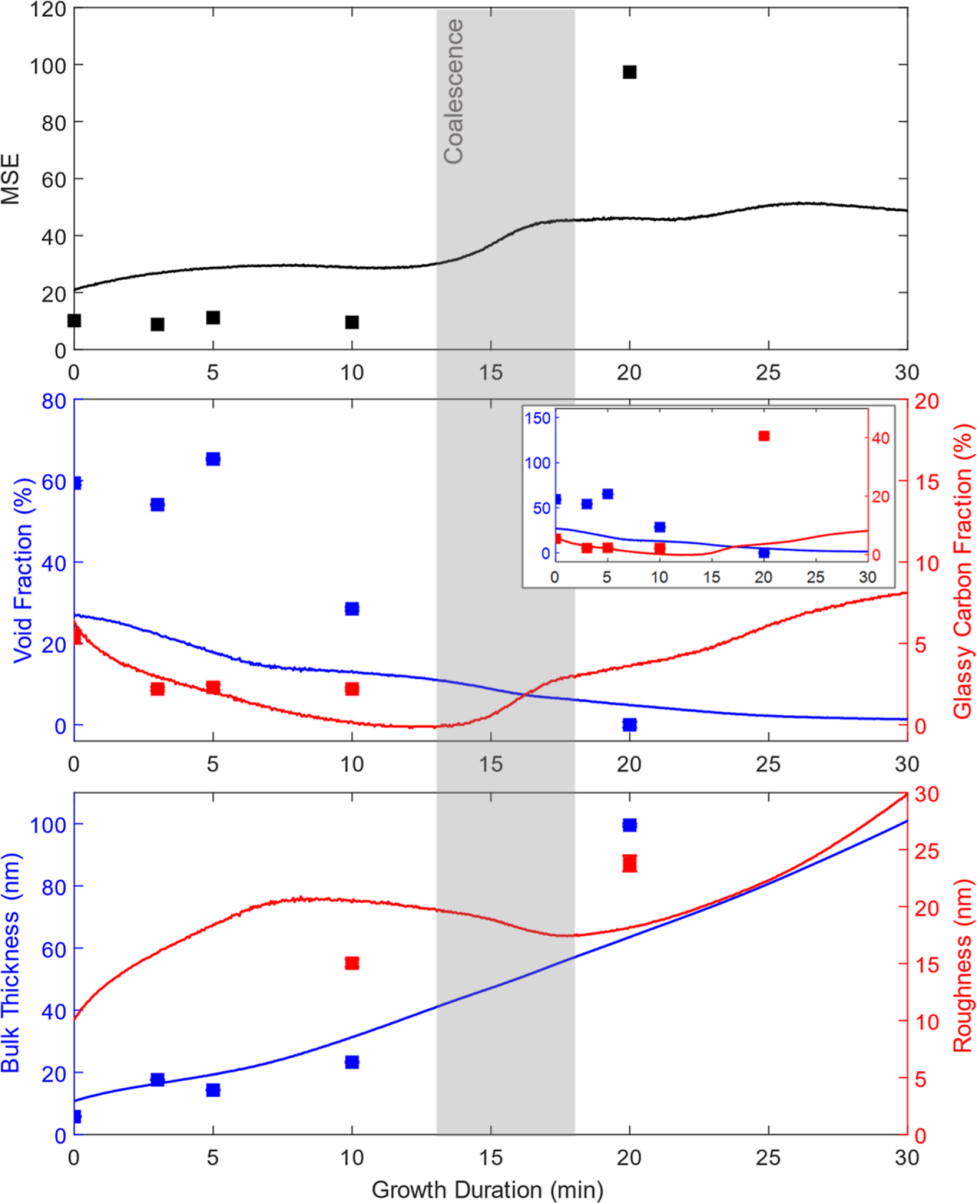
In situ and ex situ parameters. Top: MSE. Middle: SE-derived glassy carbon and void fractions. Inset: Zoomed-out view. Bottom: SE-derived bulk thickness and roughness layer thickness. Parameters from ex situ spectra are plotted as individual points, with the parameters from in situ spectra plotted as lines.

Figure 4 shows in situ SE-derived parameters for the initial 30 min of the 90 min growth duration sample, along with ex situ measurements taken from samples of varying growth duration. In the first 10 min of growth, a decrease in sp^2^ content was seen, typical of the preferential etching of non-diamond carbon seen in hydrogen-containing plasmas.^41^ At the same time, a reduction in void fraction was observed due to the Volmer–Weber growth of individual diamond nuclei. At approximately 13 min, a sharp increase in the sp^2^ of the bulk layer is seen. This is due to non-diamond carbon becoming trapped in grain boundaries as islands coalesce into a film.^17,21,24^ A peak in surface roughness was seen prior to coalescence as individual islands reached their maximum size while still remaining isolated.^24^ After coalescence, growth proceeds by the van der Drift mechanism, with overgrowth of competing crystallites leading to increased surface roughness with longer growth duration.^42^ This increasing surface roughness does result in increases in MSE further into the growth process due to depolarization, limiting SE characterization to early-stage growth.

As seen previously in SE characterization of diamond film growth on silicon,^16^ fitting of ex situ spectra resulted in a higher void content than in situ spectra, although the same trend of decreasing void fraction with growth duration is seen. This is likely the result of samples being cooled in a solely hydrogen-containing plasma, which can result in etching.^41^ While results from fitting ex situ spectra of a sample grown for 20 min are included for completeness, the very high MSE between measured and modeled spectra means that parameters derived from the SE model may not accurately reflect the true nature of this specific sample.

### 3.4 Raman Spectroscopy

**Figure 5:**
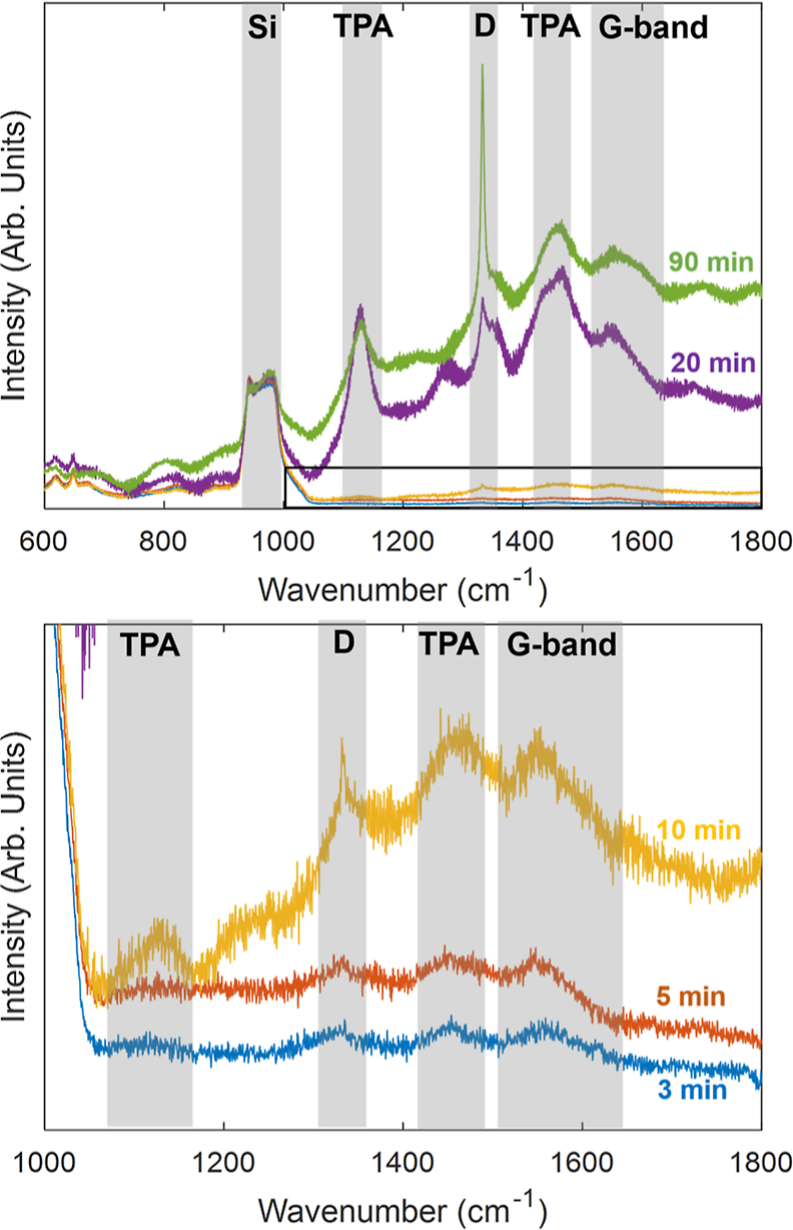
Above: Ex situ Raman spectra of samples grown for 3–90 min, normalized to the second-order silicon Raman peak. Below: Enlarged view of the region of the spectrum indicated above.

Figure 5 shows the Raman spectra of samples grown for 3–90 min. These spectra are normalized to the second-order Raman peak of silicon at 950 cm^–1^. The limited diamond film and AlN layer thicknesses mean that the most prominent peaks are from the Si substrate. These are the first- and second-order Si peaks, seen at 520 and 980 cm^–1^, respectively.^43^ Local vibrational modes of boron atoms in the doped Si substrate produce the minor peaks at 620 and 644 cm^–1^.^44,45^ At growth durations under 10 min, the first-order diamond Raman peak at 1332.5 cm^–1^^46^ is not visible due to the lower bulk diamond fraction and thickness in these samples. At 10 min, SE measurements suggest a greater bulk diamond fraction and thickness, which is matched by the appearance of the diamond Raman peak. As is typical of samples with small crystallite sizes, this peak exhibits broadening.^46,47^ Also appearing for the first time in this sample are peaks at 1140 and 1450 cm^–1^, which are assigned to *trans*-polyacetylene (TPA),^48^ in addition to the G-band at around 1550 cm^–1^ caused by in-plane stretching of pairs of sp^2^ sites.^46^ While the G peak, which is produced by the bond stretching of pairs of sp^2^ atoms in rings and chains, is typically visible at around 1560 cm^–1^,^49^ it is occluded by the G-band in this case.

At 20 min, a significant increase in the intensity of the diamond peak is observed due to the increase in both thickness and diamond content by this point. Similar increases in the intensity of the G-band and TPA peaks were seen, with the intensities of those also increasing relative to those of the diamond peak. The latter is indicative of the increase in sp^2^ content caused by the formation of grain boundaries and suggests that the coalescence of islands into a film occurs prior to 20 min, which is consistent with parameters from the in situ SE model. Seen for the first time in this sample is the D peak at 1350 cm^–1^, caused by the breathing mode of graphitic rings.^49^

The intensity of the diamond peak is higher in the 90 min growth sample due to its increased thickness. Additionally, the TPA peaks and G-band are lower in intensity relative to the diamond peak, indicating a decrease in sp^2^ content relative to the 20 min sample because of the larger crystallite size of the thicker film. The sharp diamond peak seen in this sample is indicative of a high-quality polycrystalline diamond film.

### 3.5 Atomic Force Microscopy

**Figure 6:**
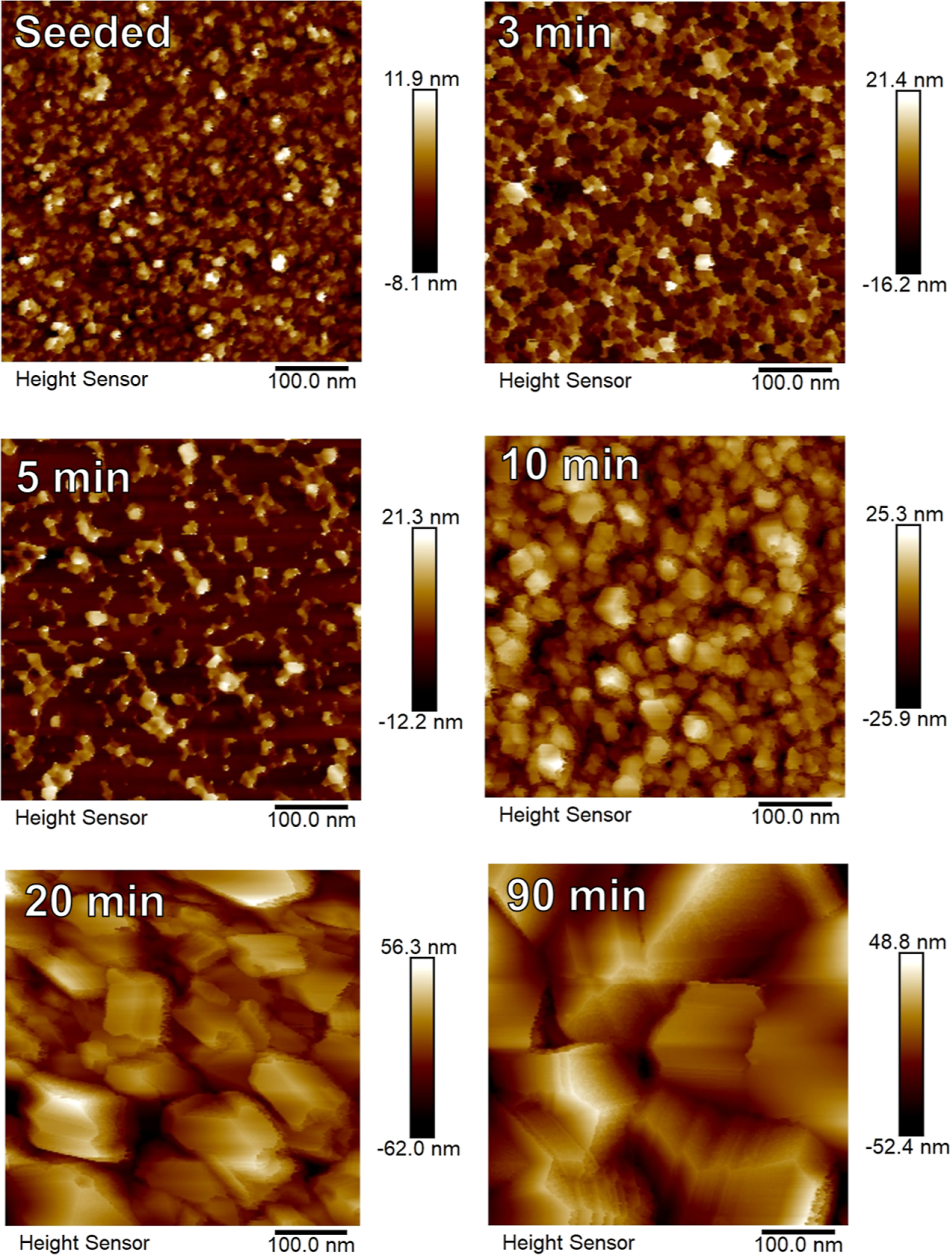
AFM images of samples with varying growth duration.

**Figure 7:**
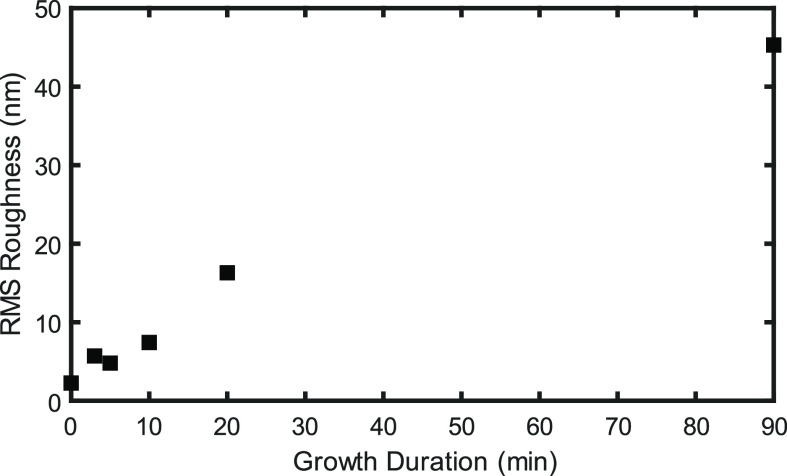
AFM-measured rms roughness of samples with varying growth duration.

Figure 6 shows AFM images of samples with varying growth durations, while Figure 7 shows the AFM-measured root mean square (rms) roughness of these samples. Up to 10 min, a gradual increase in crystallite size and rms roughness is seen. Between 10 and 20 min, a significant increase in crystallite size and roughness is seen due to coalescence and the switch to van der Drift type growth. This is another indication that coalescence occurs between 10 and 20 min, as the in situ SE model suggests. Crystallite size further increases with growth duration due to the overgrowth of crystallites, with the largest crystallite size seen in the 90 min growth duration sample.

## 4 Conclusions

In situ SE was used to investigate the initial stages of diamond growth on AlN. An optical model of the substrate and diamond film was developed from ex situ spectra, with parameters allowed to vary with the fitting process to minimize MSE between measured and modeled spectra. This model was adapted for application to in situ data and compared to ex situ Raman spectra and AFM images of samples following growth. An initial reduction in bulk layer void fraction was seen, followed by an increase in sp^2^ fraction indicative of the formation of grain boundaries during the coalescence of islands into a film. This peak in sp^2^ content was seen in Raman spectra, with an increase in intensity of peaks assigned to TPA, which is found in grain boundaries. AFM images were also consistent with the SE model, showing increasing crystallite size and rms roughness with growth duration.

Although this work was carried out on AlN films grown on silicon, the results remain applicable to films grown on an AlN layer atop a GaN device stack. The nature of the interface between a diamond film and a substrate is heavily dependent on the seeding and nucleation, which are influenced by the zeta potential of the top surface.^50^ The only impact from sub-surface layers is through differences in thermal expansion coefficient. While this may impact the stress at the interface, it will not affect the interfacial region of the diamond film.

The development of in situ characterization using SE allows monitoring of the formation of the defective interfacial layer during growth. This is critical to optimize growth parameters to reduce the thickness and sp^2^ content of this layer, reducing the thermal barrier between the AlN substrate and the diamond film and improving the effectiveness of diamond thermal management layers.

## Data Availability

Information on the data underpinning the results presented here, including how to access them, can be found in the Cardiff University data catalogue at http://doi.org/10.17035/d.2023.0277245763.

## Abbreviations

GaN: gallium nitride
AlN: aluminum nitride
SE: spectroscopic ellipsometry
MPECVD: microwave plasma-enhanced chemical vapor deposition
AFM: atomic force microscopy
MSE: mean square error
EMA: effective medium approximation
TPA: *trans*-polyacetylene
rms: root mean square
